# Supplementary material for: A qualitative study of patients’ feedback about Outpatient Parenteral Antimicrobial Therapy (OPAT) services in Northern England: implications for service improvement
Source: BMJ Open. 2018 Jan 10;8(1):e019099. doi: 10.1136/bmjopen-2017-019099 (PMC5781150; doi:10.1136/bmjopen-2017-019099)
Supplement: Author's manuscript [file bmjopen-2017-019099.draft_revisions.pdf]

BMJ Open

BMJ Open is committed to open peer review. As part of this commitment we make the peer review history of every article we publish publicly available.

When an article is published we post the peer reviewers' comments and the authors' responses online. We also post the versions of the paper that were used during peer review. These are the versions that the peer review comments apply to.

The versions of the paper that follow are the versions that were submitted during the peer review process. They are not the versions of record or the final published versions. They should not be cited or distributed as the published version of this manuscript.

BMJ Open is an open access journal and the full, final, typeset and author-corrected version of record of the manuscript is available on our site with no access controls, subscription charges or pay-per-view fees (http://bmjopen.bmj.com).

If you have any questions on BMJ Open's open peer review process please email editorial.bmjopen@bmj.com

BMJ Open

Listening to patients' feedback about Outpatient Parenteral Antimicrobial Therapy (OPAT) services – implications for service improvement

Journal:	*BMJ Open*
Manuscript ID	bmjopen-2017-019099
Article Type:	Research
Date Submitted by the Author:	10-Aug-2017
Complete List of Authors:	Twiddy, Maureen; University of Leeds, Leeds Institute of Health Sciences; University of Hull, Institute of Clinical and Applied Health Research<br>Czoski Murray, Carolyn; University of Leeds, Leeds Institute of Health Sciences<br>Mason, Samantha; University of Leeds, Institute of Cancer and Pathology<br>Meads, David; University of Leeds, Leeds Institute of Health Sciences<br>Wright, Judy; University of Leeds, Leeds Institute of Health Sciences<br>Mitchell, Elizabeth; University of Hull, Institute of Clinical and Applied Health Research<br>Minton, Jane; Leeds Teaching Hospitals NHS Trust,
**Primary Subject Heading**:	Infectious diseases
Secondary Subject Heading:	Qualitative research, Health services research
Keywords:	QUALITATIVE RESEARCH, OUTPATIENT PARENTERAL ANTI-MICROBIAL THERAPY, OPAT, PATIENT EXPERIENCES, HEALTH SERVICES PROVISION

SCHOLARONE™
Manuscripts

For peer review only

Listening to patients' feedback about Outpatient Parenteral Antimicrobial Therapy (OPAT) services – implications for service improvement

Running title: OPAT: patient experiences

Authors: Twiddy Maureen[1], Czoski Murray Carolyn, Mason Samantha, Meads David[1], Wright Judy[1], Mitchell Elizabeth D[4], Minton Jane[3]

[1]Leeds Institute of Health Sciences, University of Leeds, Leeds, UK

[2] Institute for Transport Studies, University of Leeds, Leeds, UK

[3] Leeds Teaching Hospitals NHS Trust, Leeds, UK

[4] Hull York Medical School, University of Hull, Hull HU6 7RX

Corresponding author:

Dr Maureen Twiddy, Senior Research Fellow,

Leeds Institute of Health Sciences,

Level 11, Worsley Building,

University of Leeds

Leeds

SL2 9NL

m.twiddy@leeds.ac.uk

T: +44(0)1133430830

F: +44(0)1133436997

For peer review only

Key words: OPAT, Qualitative, Patient Experiences, Home Infusion Therapy, Health Service Provision,

3250 words (excluding title page, abstract, references and tables)

For peer review only

ABSTRACT

Objective: Outpatient Parenteral Antimicrobial Therapy (OPAT) provides opportunities for improved cost-savings, but in the UK implementation is patchy and a variety of service models are in use. The slow uptake in the UK and Europe is due to a number of clinical, financial, and logistical issues, including concern about patient safety. The measurement of patient experience data is commonly used to inform commissioning decisions, but these focus on functional aspects of services, and fail to examine the relational aspects of care. This qualitative study examines patients' experiences of OPAT to understand what patients' value about the care they receive, and how services can be improved.

Design: In-depth, semi-structured interviews.

Setting: Purposive sample of OPAT patients recruited from four acute NHS Trusts in Northern England. These NHS Trusts between them represented both well established and recently set-up services running nurse at home, hospital outpatient and/or self-administration models.

Participants: We undertook 28 semi-structured interviews and one focus group (n=4).

Results: Both relational and functional aspects of patient experience matter. Services are generally able to deliver the *functional aspects of care:* delivery of OPAT care; timely management of symptoms; a safe and secure environment. However, services were often configured in ways which hamper patient access and engagement with care. *Relational aspects of care* such as co-ordination and continuity of care; and communication between patients and staff could easily be compromised, and for more vulnerable patients OPAT can be anxiety provoking.

For peer review only

Conclusion: Services configurations must accommodate the diversity of the local population. Poor communication can leave patients lacking the confidence needed to be a competent collaborator in their own care, and affect their perceptions of the service.

STRENGTHS AND LIMITATIONS OF THIS STUDY

- We recruited from four diverse sites, which enabled us to contrast the views of those who experienced different models of care and provide contextualised information.
- A relatively large qualitative sample (n=32) patients were interviewed and our broad sampling strategy meant we obtained views from participants from a diverse range of socio-economic backgrounds.
- The views of the very elderly and those from ethnic minority groups are not well represented.
- Data collection continuing after data saturation was reached.

For peer review only

INTRODUCTION

In recent years UK health policy has used measures of patient experience to identify strengths and weaknesses of service provision to inform commissioning, determine resource allocation, and drive up quality[1]. Across a range of clinical conditions, studies have found positive associations between patient experience and a range of quality indicators, including patient safety and clinical effectiveness [2 3] However, the type of data collected by these surveys do not help us understand what matters to patients[4], and tells us little about what good care looks like, or why patients experience poor care. This study explores patients' experiences of outpatient parenteral antimicrobial therapy services (OPAT), to identify key issues that affect patient experience and satisfaction.

OPAT allows patients to be given intravenous antibiotics while living in the community, rather than as a hospital in-patient.[5] It can be used for patients with a range of infections, but most commonly used for complicated soft tissue infections, bone and joint infections, endocarditis and bacteraemia.[6] Although standard practice in many countries [7 8] uptake of OPAT in the UK has been slow[9], and hampered by a range of clinical, financial, and logistical issues, not least the lack of a national commissioning strategy to support its expansion.[10]

Three service models can be used to deliver OPAT: outpatient/ambulatory care centres; a nurse visiting the patient at home; or the patient/carer trained to self-administer. With a national focus on efficiency savings and improving patient experience, OPAT is becoming more popular.[9] It is therefore important to understand patient experiences of different OPAT services to inform commissioning decisions.

What is already known?

For peer review only

Evidence regarding patient experiences of OPAT has been largely collected using patient satisfaction surveys, and there is little qualitative research to illuminate patients' experiences of these services. The results of our systematic review of OPAT services[11] found patients identified a range of benefits such as the comfort of the home environment [12-15] and increased freedom and autonomy[12 14 16-18], but not all patients view OPAT positively, with safety concerns reported.[19] There is also evidence to suggest that the information needs of OPAT patients may not always be addressed and some may find OPAT anxiety provoking.[12 15] This suggests that both functional and relational aspects of patient experience are important, but the dearth of evidence makes it difficult to draw conclusions on what good and poor care look like.

This study sought to understand patient experiences of OPAT to identify what was important to them and is part of a larger programme of work.[20]

METHOD

Design: Semi structured interviews and focus groups.

Study settings: Four hospitals in Northern England were purposively selected as they offered the following three care pathways: hospital outpatient attendance, nurse at home, and self-administration (table 1).

Table 1: Local Site Characteristics.

Site	Population size (,000)	Services provided
Teaching Hospital	500	Well established hospital attendance, visiting general nurse, and self- administration.
Teaching	800	Well established outpatient attendance and self-

For peer review only

Hospital		administration. New visiting specialised nurse service.
District General Hospital	330	Well established visiting specialised nurse, outpatient attendance, and self- administration.
District General Hospital	385	Newly established service offering visiting general nurse and outpatient attendance.

For peer review only

Participants

A purposive sampling strategy identified two groups; patients requiring short term intravenous (IV) antimicrobials (< 7 days: n=15) and patients with deep-seated infections requiring longer term IV antimicrobials > 14 days; n=25). The sample size assumed those on longer term antimicrobials represented a wider range of infections. A sampling frame was developed to capture variation in age, gender and socio-economic status. Initially, focus groups were planned but these proved difficult to recruit to so interviews were offered.

Ethical Approval Ethical approval was sought and obtained from NRES Committee South West – Frenchay (13/SW/0060).

Procedure

Interviews took place at the patient's home or the university. The focus group took place on NHS premises. Written informed consent was obtained for all participants, and discussions audio-recorded, with permission. One participant refused to be recorded (notes were taken). Interviews lasted between 30 and 75 minutes; the focus group lasted 95 minutes.

Topic Guide

The topic guide covered three questions, with probes used to explore issues in more detail. The topic guide was initially piloted on three patients and no changes made:

- What has been your experience of OPAT? What were the good and bad points in the care/service you received?
- What are the most important aspects of IV antibiotic services for you?
- If you were designing a service to provide community antibiotic IV services what would it look like?

Data analysis

Audio recordings were transcribed verbatim, anonymised and managed using NVivo10 software.[21] Data were analysed thematically to understand what patients valued about OPAT and to explore differences in their experiences.[22] Two researchers (MT, SM) independently read and coded the first three interviews. This became the initial coding frame. Codes were sorted into categories based on how they relate to one another, and themes formed. The research team agreed the coding index which was then applied to the remaining transcripts by one researcher (SM). Data saturation was reached as no new ideas were identified from the last five interviews.[23] Interview transcripts were requested by three participants; no requests for changes were received. The following notation is used in the quotes […] = text omitted. Quotes indicate participant gender, age group, course of anti-microbial (short term/ long term) and model of care experienced (nurse at home, self-administration, and hospital outpatient clinic).

RESULTS

A total of 41 patients consented. Nine subsequently declined participation pre-interview due to illness or could not be contacted. One focus group (four participants) and 28 interviews

For peer review only

took place. One interview was not used as the participant did not recall having OPAT. Demographic details are in table 2.

Table 2 Participant Demographics

	N=32
**Age**	Mean = 53 years (range 21- 80)
**Gender= Male**	16
**Marital status**	
Married	16
Single	7
Divorced/Separated/widowed	3
Co-habiting/civil partnership	6
**Ethnicity**	
White British	29
White European	2
Other (not stated)	1
**Education**	
University/professional qualification	14
College	9
Secondary	7
Did not complete formal education	2
**Employment**	
Full time (> 30 hours per week)	12
Part time (<30 hours per week)	4
Unable to work due to ill health	5
Retired	10
Carer	1
**Infection type**	

For peer review only

Short term/long term	20/12
**Service received**	
Hospital outpatient	14
Nurse at home	13
Self-administration	5

Delivery of care

Three main issues around delivery of OPAT were identified. These are described by four subthemes: being home, but not well; timeliness of care; location of care; is it safe?

Being home, but not well

For about a third of patients OPAT was an opportunity to be discharged from hospital earlier than would otherwise be the case. These participants believed that recovering at home would be better than being in hospital, and jumped at the chance of OPAT. However, few had realised just how difficult it would be to look after themselves at home and some felt staff should have alerted them to this possibility.

"*I never realised how tiring it would be though […] I never realised that just making a cuppa could be so tiring" Female, >65, long term, nurse at home*

I've just begun starting to pick up tasks again, I'm not quite there yet where I'm a fully functioning mum" Female <65, long term, hospital outpatient

For others, OPAT was an opportunity to avoid hospital admission, and although some people continued to work, for others the infection still took its toll on what they were able to do.

For peer review only

"All I went and did, was, go in the car, go to the hospital and come home, and I didn't go anywhere else, my family went, really, 'you're going to have to take it easy mum, you're going to have to rest up', and the first three days I felt really, really, ill, so I didn't want to go anywhere or do anything" Female, <65, short-term, hospital outpatient

Timeliness of care

Although some hospital outpatient OPAT services were managed via an appointment system, one NHS Trust ran their OPAT service from the medical admissions ward, leading to significant treatment delays. This resulted in the greatest level of complaints about the service, particularly from patients who were trying to balance going to work and treatment. However, most found hospital attendance convenient and preferable to hospital admission.

"[coming to hospital] it's better for my employers because if I waited until the doctors [GP] were open at nine o'clock, even if I could get a nine o'clock appointment, by the time I got into work it could be potentially eleven, eleven thirty, because it's still an hour's drive even after I've been seen" Male <65 short-term, hospital outpatient

For patients who self-administered, multiple treatments each day can leave them with little time to fit anything else into their day. This can erode the perceived benefit of being at home, as planning the next treatment was always at the back of their mind.

"There's no point really going out much or doing much cos you haven't got much time when you aren't having to think about getting everything sorted" Male <65 long term self-administer

Location of care

Where care is delivered was important to patients. Travelling to hospital could be challenging for those who relied on public transport, when apparently 'short' distances could result in two or three bus changes. Even travelling by car, patients found it difficult to park and parking

For peer review only

fees quickly mounted. Some patients suggested that dedicated short term parking bays, similar to those used by dialysis patients would help alleviate these issues. Cognisant of the cost of a nurse visiting them at home some patients suggested that OPAT services could be located in General Practice health centres.

"The elders and a lot of them feel, they feel a little bit more scared of the hospitals because some of them are single or widowed so they don't always have somebody to go with them to the hospital […] a local clinic would be much less stressful for them" <65 Male, short term hospital outpatient (A & E)

It was important that the OPAT model offered to patients met their needs. For those with multi-morbidities, attending the hospital daily or three times a week for treatment was viewed more negatively than being an in-patient, and for these patients a nurse at home model was necessary.

"I don't think that [clinic] would have worked, because […], I was still extremely weak, and one of the main reasons I wanted to come home was for the comfort of my own home, and my own bed, and to be able to rest and to build my strength up. To physically have to make a journey each day, un-necessarily in my eyes, because if I'd have stayed in hospital I wouldn't have had to make the journey, […] that done would have been exhausting" Female, <65, long term, specialist nurse

Is it safe?

Safety combined both functional and relational aspects of care. Concerns about infection risk are acknowledged and described by patients. Patients expressed confidence in the staff working in the service, and talked of the professionalism they had observed.

"the nurses that come out are specialist nurses who are informed about antibiotics and about lots of illnesses that they are treating, […] I felt very comfortable that they were very

For peer review only

knowledgeable about what I was experiencing and this reassured me about coping at home" Female, <65, long-term, specialist nurse

For some, the hospital was viewed as a safe place to receive their treatment because doctors were in attendance at the clinic, and for these patients, this embodied, *'a safe service'*. Although they were treated by a nurse, knowing a doctor was in attendance and able to monitor their care, was an attractive safety net, due to their perceived increased expertise.

"..so personally for me I felt like being treated at the hospital was probably the best option because there'd have been people around who could have come and had a look at me if they'd needed to" Female <65 short term, hospital outpatient

The nurse at home model was perceived to be a safe service because it minimised the risk of contracting infections such as C.Diff. or MRSA which they associated with hospital attendance. However, for a few patients the perceived benefits of hospital attendance did not entirely dispel these worries, and over a third of patients made some reference to the risks associated with MRSA.

"I just thought I would end up getting C Diff or MRSA in my leg. I don't want to be laid up and I don't feel ill." Female, <65, short term, hospital outpatient

For those patients self-administering their IV at home, the concepts of safety and risk were more complex. All were at significant risk of contracting infections due to underlying health issues, and knew being at home reduced this risk. However, one patient expressed concerns about being cared for at home because medical equipment needed to treat her breathing condition kept breaking down. Although she contacted the hospital for advice, she felt unsupported because staff could not answer her questions, and did not follow up to ensure proposed solutions had worked.

For peer review only

"No, I had no phone calls, many from me looking for advice, but none to me" Female, <65, long term, self-administer

Relational aspects of care

The relational aspects of care, such as emotional support, dignity, good communication and information were key to good quality care. These aspects of care are represented by two subthemes: communication; and continuity of care.

Communication

The need for good quality patient information and open lines of communication were essential. The nurse at home and self-administration models afforded the best opportunities to talk with staff. Whilst patients found outpatient clinic staff to be very knowledgeable and willing to answer questions, they were perceived to be very busy. Some older patients had concerns about being cared for out of hospital, but having a nurse to talk to provide the confidence needed to self-manage. Self-administration patients had formal training and access to a nurse by phone to provide on-going support which they viewed as essential and enabled them to be involved in decisions about their care.

"I'd got that attention completely for that time […]. I think being at home, having that person there who's just, you've just got their attention no matter what, you get to know them. I found them easy then to open up to, to ask questions" Female, <65, long-term, specialist nurse

However, any perceived breakdown in communication between OPAT staff- could erode confidence, and fuel patient anxiety about not being in hospital. When, on one occasion a nurse arrived not knowing they were to give an IV treatment, the patient questioned the competence of the team.

For peer review only

"She had no clue who I was really and arrived not knowing that she was supposed to bring the drugs with her, it did make me wonder about them" Female, <65, long term, district nurse

Continuity of Care

In addition to the breakdowns in continuity of care described already, a key transition in terms of patient care was at the end of IV treatment. Patients with long term infections were reviewed regularly, and seen at the end of treatment.

"I've got follow up in a month which is nice so they're keeping an eye on me, I wouldn't like it if I hadn't been" Female, <65, long term, hospital outpatient

In contrast, short term IV patients were not seen in clinic again and some were given no advice about what to do if symptoms returned. Although a discharge letter was sent to the patients' General Practitioner, few patients were aware of this and even fewer knew whose responsibility it was to organise a follow up appointment if needed. This lack of continuity of care was most evident with patients who had been cared for by a nurse at home as they had generally not seen a doctor after the initial diagnosis.

"I was left in the dark as to know what was after the IV, nothing at all. I'd rather if they said ok, make an appointment to see your doctor" Male, <65, short term, specialist nurse

For these short term IV patients the end of treatment was a key point where things could, and did, go wrong, and the lack of clarity about what should happen next caused uncertainty as patients were unsure who to contact.

"The doctor said four weeks when I saw her, but I'm more than four weeks on from seeing the doctor and it's still not entirely right so I don't know, no-one told me anything" Male <65 short term, hospital outpatient (A & E)

For peer review only

DISCUSSION

Patients identified a range of health care experiences as important to the quality of care received. Key to this was the type of staff involved and the skills needed to deliver good quality care. For some, this meant doctors being visibly involved in service delivery; for others a nurse led service was appropriate. All participants recognised that nurses' ability to recognise and respond appropriately to changes in the patient's health contributed to a positive healthcare experience. However, some patients may need support to manage in the community, such a rapid access to advice, and feedback on how they are coping, to help them develop confidence to manage effectively, if OPAT provision is to be widened.[13 18]

The perceived risk of contracting a hospital acquired infection was at the forefront of the minds of over half of patients. With the media labelling MRSA a superbug, it is not surprising that patient perceptions of the risk of contracting an infection have not yet caught up with the reality of reducing cases of MRSA.[24 25] In this study, a third of patients mentioned the risk of MRSA. More needs to be done to educate the public about the actual risk of MRSA, and how to minimise this.[24]

Most patients were provided with good written information but oral communication between patients and staff was more variable, reflecting the findings of other studies [12 15]. Positive relationships developed when staff found time to talk to patients about their treatment and understand them as people, rather than cases, and these encounters could help patients develop the confidence needed to take a more active role in their own care. Poor communication left some without the knowledge and confidence needed to be actively involved in their own care, and affected their perceptions of the service. These findings resonate with the conclusions of a recent review by Entwistle and colleagues[26] looking at the aspects of health care delivery that are most important to patients. Entwistle's study suggests that both the structure of healthcare and the social dynamics are important to the patient experience. Our findings lend support this conclusion.

For peer review only - http://bmjopen.bmj.com/site/about/guidelines.xhtml

Strengths and Limitations

Our data support and develops the previously limited qualitative research evaluating OPAT services. OPAT can allow patients to receive care in the community but can negatively impact on patient satisfaction if not configured to the local population. A strength of this study is that we recruited from four diverse sites, which enabled us to contrast the views of those who experienced different models of care and provide contextualised information. The results of this study were used to construct a survey to assess patient preferences for services (discrete choice experiment).[10 27] A broad sampling strategy was used to obtain views from participants from a diverse range of socio-economic backgrounds. However, we struggled to recruit the very elderly and those from ethnic minority groups. We planned to undertake focus groups but recruitment was poor, so we switch to interviews which resulted in the data collection continuing after data saturation was reached, and so no new findings were revealed in the final five interviews.

Conclusion

Nationally and internationally, healthcare organisations have highlighted the importance of patients' experiences of the services they receive, and indeed the NHS Operating Framework for England (2011) describes each patient's experience as *"the final arbiter of everything the NHS does"*.[28] In the current drive to have patients cared for in the community it is important to ensure that services are designed in a way which meet the needs of the local community to improve patient's experiences of healthcare delivery.[29] This study shows poor communication in particular can leave patients lacking the confidence needed to be a competent collaborator in their own care, and affect their perceptions of the service.

It is therefore important to understand what aspects of service provision are most important to the patient, in order to improve services.

For peer review only

ACKNOWLEGEMENTS

The authors would like to thank the rest of the CIVAS research team (Stephane Hess, Claire Hulme, David (Theo) Raynor, Rachel Vincent, Philip Stanley, Kate McLintock, and Angela Gregson), our Patient and Public Involvement group, and all the sites for their support making this research project a success.

CONFLICTS OF INTEREST

No conflicts of interest to declare.

FUNDING

This work is supported by the National Institute for Health Research HS & DR Programme. (Grant number 11/2003/60)

CONTRIBUTORSHIP STATEMENT

MT, CCM, DM and JM designed the study. MT and SM undertook the data collection and interpreted the data. EDM and JW undertook the literature searches supporting the study. All authors participated in writing the manuscript, and read and approved the final version.

DATA SHARING No additional data is available from this study.

REFERENCES

1. Clarke M, Determann D, Petrou S, et al. Discrete choice experiments in health economics: a review of the literature. Pharameconomics 2014;**32**(9):883-902.
2. Minton J, Czoski Murray C, Mead D, et al. The Community IntraVenous Antibiotic Study (CIVAS): a mixed-methods evaluation of patient preferences for and cost-effectiveness of different service models for delivering outpatient parenteral antimicrobial therapy. Health Services and Delivery Research 2017;**5**(6).
3. Doyle C, Lennox L, Bell D. A systematic review of evidence on the links between patient experience and clincial safety and effectiveness. BMJ open respiratory research 2013;**3**:e001570.
4. Doyle C, Lennox L, Bell DA. A systematic review of evidence on the links between patient experience and clinical safety and effectiveness. BMJ open 2013;**3**(e001570).

For peer review only

5. Manary MP, Boulding W, Staelin R, et al. The Patient Experience and Health Outcomes. New England Journal of Medicine 2012;**368**(3):201-03.
6. Nairn S. Emergency care and narrative knowledge. J Adv Nurs 2004;**48**:59-67.
7. Nathwani D, Morrison J, Seaton RA, et al. Out-patient and home-parenteral antibiotic therapy (OHPAT): evaluation of the impact of one year's experience in Tayside. Health Bull (Edinb) 1999;**57**(5):332-37.
8. Chary A, Tice A, Martinelli L, et al. Experience of infectious diseases consultants with outpatient parenteral antimicrobial therapy: results of an emerging infetions network survey. Clinical Infectious Deiseases 2006;**43**:1290-95.
9. Tice AD, Rehm SJ. Practice guidelines for outpatient parenteral antibiotic therapy. IDSA guidelines. Clinical Infectious Deiseases 2004;**38**:1651-72.
10. Howden BP, Grayson ML. Hospital-in-the-home treatment of infectious diseases. Med J Aust 2002;**176**:440-45.
11. Chapman AL. Outpatient parenteral antimicrobial therapy. BMJ (Clinical research ed) 2013;**346**:f1585.
12. Mitchell ED, Czoski Murray C, Meads D, et al. Clinical and cost-effectiveness, safety and acceptability of community intravenous antibiotic service models: CIVAS systematic review. BMJ (under review) 2016.
13. Fernandez-Aviles F, Carreras E, Urbano-Ispizua A, et al. Case-control comparison of at-home to total hospital care for autologous stem-cell transplantation for hematologic malignancies. Journal of Clinical Oncology 2006;**24**(30):4855-61.
14. Montalto M. Patients' and carers' satisfaction with hospital-in-the-home care. Int J Qual Health Care 1996;**8**(3):243-51.
15. Parker S, Nathwani D, O'reilly D, et al. Evaluation of the impact of non-inpatient iv antibiotic treatment for acute infections on the hospital, primary care services and the patient. J Antimicrob Chemother 1998;**42**(3):373-80.
16. Dubois A, Santos-Eggimann B. Evaluation of patients' satisfaction with hospital-at-home care. Evaluation & the Health Professions 2001;**24**(1):84-98.
17. Johansson E, Bjorkholm M, Wredling R, et al. Outpatient parenteral antibiotic therapy in patients with haematological malignancies. A pilot study of an early discharge strategy. Support Care Cancer 2001;**9**(8):619-24.
18. Kieran J, O'Reilly A, Parker J, et al. Self-administered outpatient parenteral antimicrobial therapy: a report of three years experience in the Irish healthcare setting. Eur J Clin Microbiol Infect Dis 2009;**28**(11):1369-74.
19. Martel A. Home intravenous self-injection of antibiotic therapy. CANADIAN JOURNAL OF INFECTIOUS DISEASES 1994;**5**:51C-51C.
20. Bamford KB, Desai M, Aruede MJ, et al. Patients' views and experience of intravenous and oral antimicrobial therapy: room for change. Injury 2011;**42 Suppl 5**:S24-27.
21. Czoski Murray C, Twiddy M, Meads D, et al. Community IntraVenous Antibiotic Study (CIVAS): protocol for an evaluation of patient preferences for and cost-effectiveness of community intravenous antibiotic services. BMJ open 2015;**5**(8):e008965.
22. NVivo [program]. 10 version: QSR International Pty Ltd., 2010.
23. Braun V CV. Using thematic analysis in psychology. Qualitative Research in Psychology 2006;**3**(2):77-101.
24. Morse J. The significance of saturation. Qualitative health research 195;**5**:147-49.
25. Gill J, Kumar R, Todd J, et al. Methicillin-resistant Staphylococcus aureus: awareness and perceptions. Journal of Hospital Infection 2006;**62**(3):333-37.
26. Gould DJ, Drey NS, Millar M, et al. Patients and the public: knowledge, sources of information and perceptions about healthcare-associated infection. Journal of Hospital Infection 2009;**72**(1):1-8.

For peer review only

27. Entwistle V, Firnigl D, Ryan M, et al. Which experiences of health care delivery matter to service users and why? A critical interpretive synthesis and conceptual map. Journal of health services research & policy 2012;**17**(2):70-78.
28. Department of Health. Operational guidance to the NHS – extending patient choice of provider, 2011.
29. Ziebland S, Hunt K. Using secondary analysis of qualitative data of patient experiences of health care to inform health services research and policy. J Health Serv Res Policy 2014;**19**(3):177-82.

For peer review only

BMJ Open

Listening to patients' feedback about Outpatient Parenteral Antimicrobial Therapy (OPAT) services – implications for service improvement

Journal:	*BMJ Open*
Manuscript ID	bmjopen-2017-019099.R1
Article Type:	Research
Date Submitted by the Author:	02-Nov-2017
Complete List of Authors:	Twiddy, Maureen; Univeristy of Hull, Institute of Clinical and Applied Health Research, Hull York Medical School; University of Leeds, Leeds Institute of Health Sciences<br>Czoski Murray, Carolyn; University of Leeds, Leeds Institute of Health Sciences<br>Mason, Samantha; University of Leeds, Institute of Cancer and Pathology<br>Meads, David; University of Leeds, Leeds Institute of Health Sciences<br>Wright, Judy; University of Leeds, Leeds Institute of Health Sciences<br>Mitchell, Elizabeth; University of Hull, Institute of Clinical and Applied Health Research<br>Minton, Jane; Leeds Teaching Hospitals NHS Trust,
**Primary Subject Heading**:	Infectious diseases
Secondary Subject Heading:	Qualitative research, Health services research
Keywords:	QUALITATIVE RESEARCH, OUTPATIENT PARENTERAL ANTI-MICROBIAL THERAPY, OPAT, PATIENT EXPERIENCES, HEALTH SERVICES PROVISION

SCHOLARONE™
Manuscripts

Listening to patients' feedback about Outpatient Parenteral Antimicrobial Therapy (OPAT) services – implications for service improvement

Running title: OPAT: patient experiences

Authors: Twiddy Maureen[1], Czoski Murray Carolyn[2,] Mason Samantha[4,] Meads David[2], Wright Judy[2], Mitchell Elizabeth D[1], Minton Jane[3] on behalf of the CIVAS study team.

[1] Hull York Medical School, University of Hull, Hull HU6 7RX

[2]Leeds Institute of Health Sciences, University of Leeds, Leeds, UK

[3] Leeds Teaching Hospitals NHS Trust, Leeds, UK

[4] Leeds Institute of Cancer and Pathology, University of Leeds, Leeds, UK

Corresponding author:

Dr Maureen Twiddy, Senior Lecturer,

Institute of Clinical and Applied Health Research

Hull York Medical School

University of Hull

HULL

HU6 7RX

Telephone: 01482 463279

Email: maureen.twiddy@hyms.ac.uk

Key words: OPAT, Qualitative, Patient Experiences, Home Infusion Therapy, Health Service Provision,

4358 words (excluding title page, abstract, references and tables)

For peer review only

ABSTRACT

Objective: Outpatient Parenteral Antimicrobial Therapy (OPAT) provides opportunities for improved cost-savings, but in the UK implementation is patchy and a variety of service models are in use. The slow uptake in the UK and Europe is due to a number of clinical, financial, and logistical issues, including concern about patient safety. The measurement of patient experience data is commonly used to inform commissioning decisions, but these focus on functional aspects of services, and fail to examine the relational aspects of care. This qualitative study examines patients' experiences of OPAT.

Design: In-depth, semi-structured interviews.

Setting: Purposive sample of OPAT patients recruited from four acute NHS Trusts in Northern England. These NHS Trusts between them represented both well established and recently set-up services running nurse at home, hospital outpatient and/or self-administration models.

Participants: We undertook 28 semi-structured interviews and one focus group (n=4).

Results: Despite good patient outcomes, experiences were coloured by patients' personal situation and material circumstances. Many found looking after themselves at home more difficult than they expected, whilst others continued to work despite their infection. Expensive car parking, late running services and the inconvenience of waiting in for the nurse to arrive frustrated patients, whilst efficient services, staffed by nurses with the specialist skills needed to manage IV treatment had the opposite effect. Many patients felt a local, GP or community health centre based service would resolve many of the practical difficulties that made OPAT inconvenient. Patients could find OPAT anxiety provoking but this could be ameliorated by staff taking the time to reassure patients, and provide tailored information.

For peer review only

Conclusion: Services configurations must accommodate the diversity of the local population. Poor communication can leave patients lacking the confidence needed to be a competent collaborator in their own care, and affect their perceptions of the service.

STRENGTHS AND LIMITATIONS OF THIS STUDY

- We recruited from four diverse sites, which enabled us to contrast the views of those who experienced different models of care.
- A relatively large qualitative sample (n=32) patients were interviewed and our broad sampling strategy meant we obtained views from participants from a diverse range of socio-economic backgrounds.
- The views of the very elderly and those from ethnic minority groups are not well represented.
- Data collection continued after data saturation was reached.

For peer review only

INTRODUCTION

OPAT allows patients to be given intravenous antibiotics while living in the community, rather than as a hospital in-patient. It can be used for patients with a range of infections, but most commonly used for complicated soft tissue infections, bone and joint infections, endocarditis and bacteraemia[1] Although standard practice in many countries, uptake of OPAT in the UK has been slower[1 2], and hampered by a range of clinical, financial, and logistical issues, not least the lack of a national commissioning strategy to support its expansion.[2 3]

Three service models can be used to deliver OPAT: outpatient/ambulatory care centres; a nurse visiting the patient at home; or the patient/carer trained to self-administer. With a national focus on efficiency savings and improving patient experience, OPAT is becoming more popular.[1] It is therefore important to understand patient experiences of different OPAT services to inform commissioning decisions.

In recent years UK health policy has used measures of patient experience to identify strengths and weaknesses of service provision to inform commissioning, determine resource allocation, and drive up quality[4]. Across a range of clinical conditions, studies have found positive associations between patient experience (defined by NICE and the Institute of Medicine as the relational and functional aspects of care) [5 6] and a range of quality indicators, including patient safety and clinical effectiveness. [7 8] However, the type of data collected by these surveys do not help us understand what matters to patients[9], and tells us little about what good care looks like. This study explores patients' experiences of outpatient parenteral antimicrobial therapy services (OPAT), to identify issues that affect patient experience and satisfaction.

For peer review only

What is already known?

Evidence regarding patient experiences of OPAT has been largely collected using patient satisfaction surveys, and there is little qualitative research to illuminate patients' experiences of these services. The results of our systematic review of OPAT services[10] found patients identified a range of benefits such as the comfort of the home environment and increased freedom and autonomy, but not all patients view OPAT positively, with safety concerns reported.[11] There is also evidence to suggest that the information needs of OPAT patients may not always be addressed and some may find OPAT anxiety provoking.[11] This suggests that both functional and relational aspects of patient experience are important, but the dearth of evidence makes it difficult to draw conclusions on what good and poor care look like.

This study sought to understand patient experiences of OPAT to identify what was important to them and is part of a larger programme of work. The interviews were used to develop a discrete choice experiment to examine patient preferences for services.[3] This paper constitutes a reanalysis of these data to examine patient experiences more broadly.

METHOD

Design: Semi structured interviews and focus groups.

Study settings: Four hospitals in Northern England were purposively selected as they offered the following three care pathways: hospital outpatient attendance, nurse at home, and self-administration (table 1).

Table 1: Local Site Characteristics.

Site	Population size (,000)	Services provided

For peer review only

Teaching Hospital	500	Well established hospital attendance, visiting general nurse, and self- administration.
Teaching Hospital	800	Well established outpatient attendance and self-administration. New visiting specialised nurse service.
District General Hospital	330	Well established visiting specialised nurse, outpatient attendance, and self- administration.
District General Hospital	385	Newly established service offering visiting general nurse and outpatient attendance.

For peer review only

Participants

A purposive sampling strategy identified two groups; patients requiring short term intravenous (IV) antimicrobials (< 7 days: n=15) and patients with deep-seated infections requiring longer term IV antimicrobials > 14 days; n=25). The sample size assumed those on longer term antimicrobials represented a wider range of infections. A sampling frame was developed to capture variation in age, gender and socio-economic status. Initially, focus groups were planned but these proved difficult to recruit to so interviews were offered.

Ethical Approval Ethical approval was sought and obtained from NRES Committee South West – Frenchay (13/SW/0060).

Procedure

Interviews took place at the patient's home or the university and were conducted by MT, CCM or SM (who have backgrounds in psychology, sociology and nursing). Patients were consented by nursing staff so the only contact researchers had with the participant was during the interview. The focus group was facilitated by MT and SM and took place on NHS

premises. Home interviews adhered to the University lone working policy to ensure staff safety. Written informed consent was obtained for all participants, and discussions audio-recorded, with permission. Participants were informed of their right to withdraw at any time; no participants withdrew. One participant refused to be recorded (notes were taken). Interviews lasted between 30 and 75 minutes; the focus group lasted 95 minutes.

Topic Guide

The topic guide covered three questions, with probes used to explore issues in more detail. The topic guide was initially piloted on three patients and no changes made:

- What has been your experience of OPAT? What were the good and bad points in the care/service you received?
- What are the most important aspects of IV antibiotic services for you?
- If you were designing a service to provide community antibiotic IV services what would it look like?

Data analysis

The interviews were originally conducted as part of a mixed methods study to identify attributes of care which could be used to develop a discrete choice experiment.[3] This paper provides a reanalysis of that those data to understand participant experiences and in doing so takes a subtle realist approach which accepts the social world exists independently of our understanding of it, but that it is only accessible via participants experiences and interpretations.[12]

Audio recordings were transcribed verbatim, anonymised and managed using NVivo10 software.[13] Transcripts were checked to ensure patient confidentiality was maintained and material removed from the transcript which could possibly identify individuals (e.g. name of their doctor, family members). Coding was inductive, identifying issues of importance to patients. Data were later explored using the conceptual framework developed by Entwistle

For peer review only

and colleagues.[14] Two researchers (MT, SM) independently read and coded the first three interviews. This became the initial coding frame. Codes were sorted into categories based on how they relate to one another, and themes formed. The research team agreed the coding index which was then applied to the remaining transcripts by one researcher (SM). Data saturation was reached as no new ideas were identified from the last five interviews.[15] Interview transcripts were requested by three participants; no requests for changes were received. The following notation is used in the quotes […] = text omitted. Quotes indicate participant gender, age group, course of anti-microbial (short term/ long term) and model of care experienced (nurse at home, self-administration, and hospital outpatient clinic).

RESULTS

A total of 41 patients consented. Nine subsequently declined participation pre-interview due to illness or could not be contacted. One focus group (four participants) and 28 interviews took place. One interview was not used as the participant did not recall having OPAT. The focus group participants came from one hospital and all received a nurse at home model so although the issues they identified around nurse at home care reflect the experiences of patients at other centres they did not contribute to our understanding of the other models of care. As a result the findings of the focus group and interviews were analysed with the interview data. Demographic details are in table 2.

Table 2 Participant Demographics

	N=32
**Age**	Mean = 53 years (range 21- 80)
**Gender= Male**	16
**Marital status**	
Married	16
Single	7

For peer review only

Divorced/Separated/widowed	3
Co-habiting/civil partnership	6
**Ethnicity**	
White British	29
White European	2
Other (not stated)	1
**Education**	
University/professional qualification	14
College	9
Secondary	7
Did not complete formal education	2
**Employment**	
Full time (> 30 hours per week)	12
Part time (<30 hours per week)	4
Unable to work due to ill health	5
Retired	10
Carer	1
**Infection type**	
Short term/long term	20/12
**Service received**	
Hospital outpatient	14
Nurse at home	13
Self-administration	5

Two key themes were identified which map to functional and relational aspects of care.

Functional Aspects of Care

This theme relates to the functional aspects of care which are described by four subthemes: being home, but not well; convenience and flexibility; location of care; is it safe?

Being home, but not well

For most patients OPAT was an opportunity to be discharged from hospital earlier than would otherwise be the case. These participants believed that recovering at home would be better than being in hospital, and welcomed the opportunity to try OPAT. However, few realised how difficult it would be to look after themselves at home and some felt staff should have been more alert to their personal situation and circumstances.

"*I never realised how tiring it would be though […] I never realised that just making a cuppa could be so tiring"* female, age 60-70, long term, nurse at home

I've just begun starting to pick up tasks again, I'm not quite there yet where I'm a fully functioning mum" female, age 40-50, long term, hospital outpatient

For others, OPAT was an opportunity to avoid hospital admission, and although some people continued to work, for others, the infection limited their activities.

"All I went and did, was, go in the car, go to the hospital and come home, and I didn't go anywhere else, […] the first three days I felt really, really, ill, so I didn't want to go anywhere or do anything" female, age 50-60 , short-term, hospital outpatient

Convenience and Flexibility

Although some hospital outpatient OPAT services were managed via an appointment system, one NHS Trust ran their OPAT service from the medical admissions ward, leading to significant delays which proved particularly difficult for patients who were trying to balance going to work and treatment.

For peer review only

"[I] thought 'I'll be back in work by sort of quarter to eleven', by three o'clock I still hadn't been seen, […] I didn't like that whatsoever" male, 40-50, short term, hospital outpatient

Despite having an infection serious enough to require IV treatment many working age people did not take sick leave. Some felt well, but others found it difficult because they were not viewed by their managers as 'ill enough'.

"I can't walk, booked a week and a half off work, […] then my boss rang me and was like 'I need you to work" female, age 30-40, short term, hospital outpatient

When appointment systems worked well most found hospital attendance convenient and appreciated that treatment could be fitted around their personal circumstances.

"[coming to hospital] it's better for my employers" male, age 40-50, short-term, hospital outpatient

For patients who self-administered, multiple treatments each day can leave them with little time to fit anything else into their day. Although some coped by taking their IV kit with them and infusing 'on the go', others found the perceived benefit of being at home was eroded, as planning the next treatment was always at the back of their mind.

"There's no point really going out much or doing much cos you haven't got much time when you aren't having to think about getting everything sorted" male, age 30-40,long term self-administer

Location of care

Where care is delivered was important to patients. Travelling to hospital could be challenging for those who relied on public transport, when apparently 'short' distances could result in two

For peer review only

or three bus changes. Even travelling by car, patients found it difficult to park and fees quickly mounted. Some patients suggested that dedicated short term parking bays, similar to those used by dialysis patients would help alleviate these issues. Cognisant of the cost of a nurse visiting them at home some patients suggested that OPAT services could be located in General Practice health centres. Others would have liked to visit their GP practice because they found the nurse at home model too restrictive.

“waiting in for a district nurse wasn’t something that I liked, because of the inconvenience of being tied to your home waiting for them” female, age 50-60, short term, hospital outpatient

“[older people] feel a little bit more scared of the hospitals because some of them are single or widowed so they don’t always have somebody to go with them […] a local clinic would be much less stressful for them” male, age 50-60, short term, hospital outpatient

It was important that the OPAT model offered to patients met their needs. For those with multi-morbidities, attending the hospital daily or three times a week for treatment was viewed more negatively than being an in-patient, making a nurse at home model necessary. These patients also often had multiple agencies involved in their care and so it was important to ensure they could cope in the community as they were often weakened by the effect of other conditions.

“I don’t think that [clinic] would have worked, because […], I was still extremely weak, […] To physically have to make a journey each day, un-necessarily in my eyes, because if I’d have stayed in hospital I wouldn’t have had to make the journey, […] that done would have been exhausting” female, age 40-50, long term, specialist nurse

Is it safe?

For peer review only

Safety combined both functional and relational aspects of care. Concerns about infection risk are acknowledged and described by patients. Patients expressed confidence in the staff working in the service to minimise risks, and talked of the professionalism they had observed.

“nurses […] were very knowledgeable about what I was experiencing and this reassured me about coping at home” Female, age 40-50, long-term, specialist nurse

For some, the hospital was viewed as a safe place to receive their treatment because doctors were in attendance at the clinic, and for these patients, this embodied, *‘a safe service’*. Although they were treated by a nurse, knowing a doctor was in attendance and able to monitor their care, was an attractive safety net, due to their perceived increased expertise.

“..so personally for me I felt like being treated at the hospital was probably the best option because there’d have been people around who could have come and had a look at me if they’d needed to” female, age 50-60, short term, hospital outpatient

The nurse at home model was perceived to be a safe service because it minimised the risk of contracting infections such as *Clostridium difficile* (C Diff) which they associated with hospital attendance. For a few patients the perceived benefits of hospital attendance did not entirely dispel these worries, and over a third of patients made some reference to the risks associated with methicillin-resistant *Staphylococcus aureus* (MRSA).

“I just thought I would end up getting C Diff or MRSA in my leg. I don’t want to be laid up and I don’t feel ill.” female, age 40-50, short term, hospital outpatient

For those patients self-administering their IV at home, the concepts of safety and risk were more complex. All were at significant risk of contracting infections due to underlying health issues, and knew being at home reduced this risk. For them, maintaining aseptic technique, correctly storing their medication and administering their drugs were second nature, but all

For peer review only

were aware of the consequences of any lapse of judgement and valued the reminders given by nursing staff.

"They give you a booklet every single time and go through it every time; they go through obviously your flushes, even though we've been doing it for years, cos if I get it wrong I am back in here (hospital)" female, age 20-30, long term, self-administration.

Relational aspects of care

This theme describes the relational aspects of care, such as emotional support, treating people as individuals, good communication and information which were key to good quality care. Participants gave examples of where nursing staff had reduced patients' OPAT related anxiety and distress, and explained that they did this with sensitivity and professionalism, ensuring that the patient's dignity was maintained.

"I felt, I felt quite sorry for them cause I was just having such a panic and just like, you must have to deal with crazy people all the time and they were really nice […], they didn't make a big thing of it but got me somewhere quiet" female, age 20-30, short term, hospital outpatient

All patients recalled receiving good quality written information but this was often generic and did not answer their questions; e.g. how to shower with a cannula in place, or how to get additional support at home. Some older patients had concerns about being cared for out of hospital, and described how having the nurse to talk to provided the confidence needed to self-manage.

"I'd got that attention completely for that time […]. you've just got their attention no matter what, you get to know them. I found them easy then to open up to, to ask questions" female, age 60-70, long-term, specialist nurse

Although there were many examples of good care, the presence of cannula or port to facilitate drug administration was distressing for many who had no previous experience of IV

For peer review only

administration, and patients felt that staff did not appear to acknowledge the impact this had on them, in particular the fear engendered going about everyday activities.

"They gave me, you know the cannula, they were like 'we put this is and we leave it in your arm' which made me like die a little bit inside, then the fear of it being knocked at home, that killed me" Female, age 30-40, short term, hospital outpatient

The visibility of the cannula was particularly troublesome when travelling by public transport as there was a perceived risk of injury and a fear of being judged by its presence.

"I thought y'know err what's people gonna think about this? [I was] concerned about how it would be perceived you know, wandering [about with cannula in]" Female, age 40-50, short term, hospital outpatient

In contrast, self-administration patients had formal training about IV management and access to a nurse by phone to provide on-going support which they viewed as essential and enabled them to be fully involved in decisions about their care.

A perceived breakdown in communication between OPAT staff could erode patient confidence, and fuel anxiety about not being in hospital. When, on one occasion a nurse arrived not knowing they were to give an IV treatment, the patient questioned the competence of the team. Similarly, examples were given of staff coming to remove cannula that had already been removed, or to give IV antibiotics to patients who had been switched to oral medication and were provided as incidents of poor care.

"She had no clue who I was really and arrived not knowing that she was supposed to bring the drugs with her, it did make me wonder about them" female, age 40-50, long term, district nurse

For peer review only

A key transition in terms of patient care was at the end of IV treatment. Patients with long term infections were reviewed regularly, and seen at the end of treatment, and all were satisfied with their follow-up.

"I've got follow up in a month which is nice so they're keeping an eye on me, I wouldn't like it if I hadn't been" female, age 40-50, long term, hospital outpatient

In contrast, short term IV patients were not seen in clinic again and some were given no advice about what to do if symptoms returned. Although a discharge letter was sent to the patients' General Medical Practitioner, few patients were aware of this and even fewer knew whose responsibility it was to organise a follow up appointment if needed. This lack of continuity of care was most evident with patients who had been cared for by a nurse at home as they had generally not seen a doctor after the initial diagnosis, and these patients commonly described feeling left in the dark about their future care.

"I was left in the dark as to know what was after the IV, nothing at all. I'd rather if they said ok, make an appointment to see your doctor" male, age 50-60, short term, specialist nurse

For these short term IV patients the end of treatment was a key point where things could, and did, go wrong, and the lack of clarity about what should happen next caused uncertainty as patients were unsure who to contact.

"The doctor said four weeks when I saw her, but I'm more than four weeks on from seeing the doctor and it's still not entirely right so I don't know, no-one told me anything" male, age 40-50 short term, hospital outpatient

DISCUSSION

Patients identified a range of health care experiences as important to the quality of care received. Important considerations were: being cared for in a way that fits their personal circumstances (location and type of OPAT), the type of staff involved, and staff able to

For peer review only

deliver good quality care. Where patients were cared for and by whom was important. For some, this meant doctors being visibly involved in service delivery; for others a nurse led service was appropriate. All participants recognised that nurses' ability to recognise and respond appropriately to changes in the patient's health contributed to a positive healthcare experience.

Satisfaction with OPAT services was high, a finding which is well reported in the literature.[10] However, there was also evidence that services were not always well aligned to the personal and material resources of the patient. The contextual factors that affected how well patients cope included: what support families had at home, personal circumstances (e.g. self-employed), material resources, such as car access for daily attendance at hospital and the provision of information tailored to their situation. Other studies have found families may not have the personal resources to care for a family member at home[16 17], and our findings support this conclusion.

It is recognised that patients often find being cared for out of hospital worrying, and providing access to advice can boost confidence[17 18]. However, the information needs of patients are often not met.[11] The present study supports these findings, but also suggests that even when patient outcomes are good, as was the case in this study, interpersonal relationships are important.

Most patients were provided with good written information but tailored information was absent, and oral communication between patients and staff was more variable. Positive relationships developed when staff found time to talk to patients about their treatment and understand them as people, rather than cases. These encounters could help patients develop the confidence needed to take a more active role in their own care. Poor communication left some without the knowledge and confidence needed to be actively involved in their own care, and affected their perceptions of the service. These findings resonate with the conclusions of a recent review by Entwistle and colleagues[14] looking at the

For peer review only

377 aspects of health care delivery that are most important to patients. Entwistle's study suggests that both the structure of healthcare and the social dynamics are important to the patient experience. Our findings lend support this conclusion.

380 The perceived risk of contracting a hospital acquired infection was at the forefront of the minds of many patients. With the media labelling MRSA a superbug, it is not surprising that patient perceptions of the risk of contracting an infection have not yet caught up with the reality of reducing cases of MRSA. Earlier studies have found there to be high levels of awareness of MRSA, with one study in 2006 finding 94% of patients were aware of MRSA, with 68% finding information about MRSA from the media.[19] Another qualitative study found the majority of patients had little confidence in the NHS in relation to health-care-related infections.[20] In the present study, this discourse was still prevalent and suggests more needs to be done to educate the public about the actual risk of MRSA, and how to minimise these, especially in the light of increasing drug resistance.

391 The findings of this study were used to develop a discrete choice experiment (DCE) to seek to understand patient preferences for OPAT services. The DCE was distributed to 202 people who had previous experience of OPAT and found that looking at the whole sample, patients were more likely to choose a nurse at home model over a hospital or self-administration model; there was a preference for timed appointments, and for treatment delivered by a specialist, rather than generalist nurse, and communication with someone they know. However, there was significant heterogeneity across patient types, albeit with an overall preference for the nurse at home model.[3] These findings align with our qualitative findings, and argue for flexible service as a one-size does not fit all.

400 **Strengths and Limitations**

401 Our data support and develops the previously limited qualitative research evaluating OPAT services. OPAT can allow patients to receive care in the community but can patient

For peer review only

satisfaction can be reduced if not configured to the local population. A strength of this study is that we recruited from four diverse sites[3 21], and a broad sampling strategy was used to obtain views from participants from a diverse range of socio-economic backgrounds. However, we struggled to recruit the very elderly and those from ethnic minority groups. We planned to undertake focus groups but recruitment was poor, so we switched to interviews which resulted in the data collection continuing after data saturation was reached, and so no new findings were revealed in the final five interviews.

Conclusion

Nationally and internationally, healthcare organisations have highlighted the importance of patients' experiences of the services they receive, and indeed the NHS Operating Framework for England (2011) describes each patient's experience as *"the final arbiter of everything the NHS does"* .[22] In the current drive to have patients cared for in the community it is important to ensure that services are designed in a way which meet the needs of the local community to improve patient's experiences of healthcare delivery.[23] This study shows poor communication can leave patients lacking the confidence needed to be a competent collaborator in their own care, and affect their perceptions of the service, even when they have positive health outcomes.

It is therefore important to understand what aspects of service provision are most important to the patient, in order to improve services.

ACKNOWLEGEMENTS

The authors would like to thank the rest of the CIVAS research team (Stephane Hess, Claire Hulme, David (Theo) Raynor, Rachel Vincent, Philip Stanley, Kate McLintock, and Angela Gregson), our Patient and Public Involvement group, and all the sites for their support making this research project a success.

For peer review only

CONFLICTS OF INTEREST

No conflicts of interest to declare.

FUNDING

This work is supported by the National Institute for Health Research HS & DR Programme. (Grant number 11/2003/60)

CONTRIBUTORSHIP STATEMENT

MT, CCM, DM and JM designed the study. MT, CCM and SM undertook the data collection and interpreted the data. EDM and JW undertook the literature searches supporting the study. All authors participated in writing the manuscript, and read and approved the final version.

DATA SHARING No additional data is available from this study.

REFERENCES

1. Chapman AL. Outpatient parenteral antimicrobial therapy. BMJ (Clinical research ed) 2013;**346**:f1585.
2. Nathwani D. Developments in outpatient parenteral antimicrobial therapy (OPAT) for Gram-positive infections in Europe, and the potential impact of daptomycin. J Antimicrob Chemother 2009;**64**(3):447-53.
3. Minton J, Czoski Murray C, Mead D, et al. The Community IntraVenous Antibiotic Study (CIVAS): a mixed-methods evaluation of patient preferences for and cost-effectiveness of different service models for delivering outpatient parenteral antimicrobial therapy. Health Services and Delivery Research 2017;**5**(6).
4. Doyle C, Lennox L, Bell D. A systematic review of evidence on the links between patient experience and clincial safety and effectiveness. BMJ open respiratory research 2013;**3**:e001570.
5. NICE. Patient exprience in adult NHS services: improving the experience of care for people using adult NHS services. Manchester: NICE, 2011.
6. Institute of Medicine. Crossing the quality chasm: a new health system for the 21st century. Washington, DC: National Academy Press. , 2001.
7. Doyle C, Lennox L, Bell DA. A systematic review of evidence on the links between patient experience and clinical safety and effectiveness. BMJ open 2013;**3**(e001570).
8. Manary MP, Boulding W, Staelin R, et al. The Patient Experience and Health Outcomes. New England Journal of Medicine 2012;**368**(3):201-03.
9. Nairn S. Emergency care and narrative knowledge. J Adv Nurs 2004;**48**:59-67.

For peer review only

10. Mitchell ED, Czoski Murray C, Meads D, et al. Clinical and cost-effectiveness, safety and acceptability of community intravenous antibiotic service models: CIVAS systematic review. BMJ (under review) 2016.
11. Bamford KB, Desai M, Aruede MJ, et al. Patients' views and experience of intravenous and oral antimicrobial therapy: room for change. Injury 2011;**42 Suppl 5**:S24-27.
12. Hammersley M. *What's wrong with Ethnography?* London: Routledge, 1992.
13. NVivo [program]. 10 version: QSR International Pty Ltd., 2010.
14. Entwistle V, Firnigl D, Ryan M, et al. Which experiences of health care delivery matter to service users and why? A critical interpretive synthesis and conceptual map. Journal of health services research & policy 2012;**17**(2):70-78.
15. Morse J. The significance of saturation. Qualitative health research 1995;**5**:147-49.
16. Dubois A, Santos-Eggimann B. Evaluation of patients' satisfaction with hospital-at-home care. Evaluation & the Health Professions 2001;**24**(1):84-98.
17. Montalto M. Patients' and carers' satisfaction with hospital-in-the-home care. Int J Qual Health Care 1996;**8**(3):243-51.
18. Martel A. Home intravenous self-injection of antibiotic therapy. CANADIAN JOURNAL OF INFECTIOUS DISEASES 1994;**5**:51C-51C.
19. Gill J, Kumar R, Todd J, et al. Methicillin-resistant Staphylococcus aureus: awareness and perceptions. Journal of Hospital Infection 2006;**62**(3):333-37.
20. Burnett E, Lee KB, Rushmer R, et al. Healthcare-associated infection and the patient experience: a qualitative study using patient interviews. Journal of Hospital Infection 2010;**74**:42-47.
21. Clarke M, Determann D, Petrou S, et al. Discrete choice experiments in health economics: a review of the literature. Pharameconomics 2014;**32**(9):883-902.
22. Department of Health. Operational guidance to the NHS – extending patient choice of provider, 2011.
23. Ziebland S, Hunt K. Using secondary analysis of qualitative data of patient experiences of health care to inform health services research and policy. J Health Serv Res Policy 2014;**19**(3):177-82.

For peer review only

Standards for Reporting Qualitative Research: A Synthesis of Recommendations

Bridget C. O'Brien, PhD, Ilene B. Harris, PhD, Thomas J. Beckman, MD,
Darcy A. Reed, MD, MPH, and David A. Cook, MD, MHPE

Acad Med. 2014;89:1245–1251. First published online June 20, 2014
doi: 10.1097/ACM.0000000000000388

PAPER TITLE: **Listening to patients' feedback about Outpatient Parenteral Antimicrobial Therapy (OPAT) services – implications for service improvement**

Lead Author: Twiddy, M
manuscript ID bmjopen-2017-019099.R1

	**Title and abstract**		PAGE
S1	Title	Concise description of the nature and topic of the study Identifying the study as qualitative or indicating the approach (e.g., ethnography, grounded theory) or data collection methods (e.g., interview, focus group) is recommended	1
S2	Abstract	Summary of key elements of the study using the abstract format of the intended publication; typically includes background, purpose, methods, results, and conclusions	3
	**Introduction**		
S3	Problem formulation	Description and significance of the problem/phenomenon studied; review of relevant theory and empirical work; problem statement	5
S4	Purpose or research question	Purpose of the study and specific objectives or questions	6
	**Method**		
S5	Qualitative method and research paradigm	Qualitative approach (e.g., ethnography, grounded theory, case study, phenomenology, narrative research) and guiding theory if appropriate; identifying the research paradigm (e.g., postpositivist, constructivist/ interpretivist) is also recommended; rationale[b]	8
S6	Researcher characteristics and reflexivity	Researchers' characteristics that may influence the research, including personal attributes, qualifications/experience, relationship with participants, assumptions, and/or presuppositions; potential or actual interaction between researchers' characteristics and the research questions, approach, methods, results, and/or transferability	7
S7	Context	Setting/site and salient contextual factors; rationale[b]	8
S8	Sampling strategy	How and why research participants, documents, or events were selected; criteria for deciding when no further sampling was necessary (e.g., sampling saturation); rationale[b]	7 & 8
S9	Ethical issues pertaining to human subjects	Documentation of approval by an appropriate ethics review board and participant consent, or explanation for lack thereof; other confidentiality and data security issues	7 & 8

For peer review only

S10	Data collection methods	Types of data collected; details of data collection procedures including (as appropriate) start and stop dates of data collection and analysis, iterative process, triangulation of sources/methods, and modification of procedures in response to evolving study findings; rationale[b]	8 & 9
S11	Data collection instruments and technologies	Description of instruments (e.g., interview guides, questionnaires) and devices (e.g., audio recorders) used for data collection; if/how the instrument(s) changed over the course of the study	7 & 8
S12	Units of study	Number and relevant characteristics of participants, documents, or events included in the study; level of participation (could be reported in results)	9
S13	Data processing	Methods for processing data prior to and during analysis, including transcription, data entry, data management and security, verification of data integrity, data coding, and anonymization/deidentification of excerpts	8
S14	Data analysis	Process by which inferences, themes, etc., were identified and developed, including the researchers involved in data analysis; usually references a specific paradigm or approach; rationale	8 & 9
S15	Techniques to enhance trustworthiness	Techniques to enhance trustworthiness and credibility of data analysis (e.g., member checking, audit trail, triangulation); rationale[b]	9
	**Results/findings**		
S16	Synthesis and interpretation	Main findings (e.g., interpretations, inferences, and themes); might include development of a theory or model, or integration with prior research or theory	10-19
S17	Links to empirical data	Evidence (e.g., quotes, field notes, text excerpts, photographs) to substantiate analytic findings	10-19
	**Discussion**		
S18	Integration with prior work, implications, transferability, and contribution(s) to the field	Short summary of main findings; explanation of how findings and conclusions connect to, support, elaborate on, or challenge conclusions of earlier scholarship; discussion of scope of application/ generalizability; identification of unique contribution(s) to scholarship in a discipline or field	19-21
S19	Limitations	Trustworthiness and limitations of findings	21
	**Other**		
S20	Conflicts of Interest	Potential sources of influence or perceived influence on study conduct and conclusions; how these were managed	22
S21	Funding	Sources of funding and other support; role of funders in data collection, interpretation, and reporting	22

For peer review only
